# Prognostic value of serum bilirubin in patients with heart failure: A protocol for a systematic review and meta-analysis

**DOI:** 10.1097/MD.0000000000026180

**Published:** 2021-06-04

**Authors:** Huan Wang, Qiulei Jia, Jingjing Shi, Yuanhui Hu

**Affiliations:** a Department of Cardiology, Guang’anmen Hospital, China Academy of Chinese Medical Sciences; b Graduate School, Beijing University of Chinese Medicine; c Graduate School, China Academy of Chinese Medical Sciences, Beijing, China.

**Keywords:** bilirubin, heart failure, meta-analysis, prognostic, systematic review

## Abstract

**Background:**

Heart failure (HF) is one of the common and critical disease, and often accompanied by increased level of serum bilirubin, but the role of an indicator of bilirubin to monitor the prognosis of patients with heart failure is still unclear, so we implemented the study to systematically evaluate the predictive value of bilirubin in HF.

**Methods:**

A comprehensive search and systematic review will be conducted on electronic databases such as Medline, Cochrane Library, Embase, Web of Science, Cochrane Clinical Trials Database of study on the relationship between bilirubin and prognosis of HF patients. Review Manager software (version 5.3.5) and STATA 14 software (version 14.0) will be used for data analysis and synthesis.

**Results:**

The results will systematically and comprehensively reveal the evidence on the predictive value of bilirubin in HF.

**Conclusion:**

The study will display the effect of bilirubin level on the prognosis of patients with heart failure, and help clinicians to pay more attention to the level of bilirubin in patients with HF, and can take certain treatment measures as earlier as possible.

**Inplasy registration number:**

INPLASY202140116.

## 1 Introduction

Heart failure (HF) is a complex and common clinically syndrome. There are more than 8 million of HF patients in China. The prevalence of HF in adults is about 1%, while that of people over 70 years old is more than 10%.^[1,2]^ The pathology involves in multiple organs and pathological segments. In recent years, despite the treatment of HF has made great progress, the prognosis of HF patients is still unsatisfactory. Data shows that deaths caused by HF account for 10% of all deaths in the population, and the 5-year survival rate of patients is less than 50%.^[3,4]^ Therefore, studying the factors of the risk of death in HF patients is of great significance to reduce the mortality rate and prolong survival. However, its prognostic factors are very complicated, in which that the function of serum bilirubin can not be ignored and is controversy. The level of bilirubin can predict the responsiveness of cardiac resynchronization therapy treatment in CHF patients, and its elevated level is related to the incidence of adverse cardiovascular events and mortality in CHF patients.^[5]^ The EVREST study has revealed that abnormal liver function including total bilirubin can predict cardiovascular death.^[6]^ Large-scale CHARM studies have also confirmed that elevated total bilirubin is the strongest predictor of all-cause death, cardiovascular death and admissions in HF.^[7]^ Yet serum bilirubin has the effects of anti-inflammatory, antioxidant, and endothelial functions.^[8]^ Total bilirubin is not an independent predictor of death in patients with heart failure.^[9]^

Heart failure can cause liver damage.^[10]^ There are 2 main mechanisms that cause the increase of bilirubin. One is the increased central venous pressure of patients with HF, which leads to passive liver congestion, and cause to direct and indirect bilirubin increase, all is called “congestive hepatomegaly.”^[11]^ The other one is decreased liver perfusion caused by reduced cardiac output, which can lead to acute liver cell damage and even necrosis, this is called “cardiogenic liver ischemia (also known as shock liver).”^[12]^ The former is more common in chronic heart failure, while the latter is more common in acute heart failure or acute decompensation of chronic heart failure.^[7,13]^

So there may be greater predictive value for the level of bilirubin indicating the prognosis of HF. Therefore, we intend to do a meta-analysis of the relationship between the two, aiming to explore the influence of bilirubin on the prognosis of patients with heart failure, so as to provide a reliable data support for the clinical measures.

## 2 Methods

### 2.1 Registration

This protocol has been registered on International Platform of Registered Systematic Review and Meta-Analysis Protocols (INPLASY202140116), available from https://inplasy.com/inplasy-2021-4-0116/.

### 2.2 Eligibility criteria

#### 2.2.1 Types of studies

The meta-analysis will focus on prospective trials (randomized or nonrandomized) and retrospective studies which aim to observe the prognostic value of bilirubin in patients with HF. Animal studies, comments, reviews, letters, conference abstracts, case reports, unable to obtain sufficient original data, or incomplete data as well as literature not in English will be all excluded.

#### 2.2.2 Types of participants

Participants who were more than 18 years old and were diagnosed with heart failure were the eligible objects in the study.

#### 2.2.3 Types of prognostic factors

The role of bilirubin as prognostic factors will be performed after adjusting for other possible covariates. During meta-analysis, the low and middle bilirubin groups of 2, 3, or 4 equals were defined as low bilirubin group, and the other groups were defined as high bilirubin group.

#### 2.2.4 Type of outcomes

##### 2.2.4.1 The main results

The main results will include:

1. MACE, including cardiovascular events, myocardial infarction, and stroke.
2. All-cause mortality.

We will consider the results embracing in-hospital, long-term follow-up events and all of these mortality.

##### 2.2.4.2 Secondary results

Secondary results will include:

1. Length of stay in intensive/cardiovascular intensive care unit.
2. Use of vasoactive drugs.

### 2.3 Search methods for the identification of studies

#### 2.3.1 Database retrieval

The search strategy which is more detailed and standardized is the consensus among all reviewers. As shown in Table 1, the search strategy will be used for retrieval in Medline and modified according to the different requirements of other databases. The search scope includes 5 databases including Medline, Cochrane Library, Embase, Web of Science, Cochrane Clinical Trials Database. The retrieval time is from the beginning to August 2021. Table 1 shows the detailed search strategy in Medline.

**Table 1:** The search strategy in Medline.

No.	Keywords (including MeSH words)
#1	(heart failure [MeSH Terms]) OR (Cardiac Failure) OR (Heart Decompensation) OR (Decompensation, Heart) OR (Heart Failure, Right-Sided) OR (Heart Failure, Right Sided) OR (Right-Sided Heart Failure) OR (Right Sided Heart Failure) OR (Myocardial Failure) OR (Congestive Heart Failure) OR (Heart Failure, Congestive) OR(Heart Failure, Left-Sided) OR (Heart Failure, Left Sided) OR (Left-Sided Heart Failure) OR (Left Sided Heart Failure)
#2	(Bilirubin [MeSH Terms]) OR (Bilirubin IX alpha) OR (Bilirubin, (4E)-Isomer) OR (Bilirubin, (4E,15E)-Isomer) OR (Hematoidin) OR (Bilirubin, Disodium Salt) OR (Disodium Salt Bilirubin) OR (Bilirubin, Monosodium Salt) OR (Monosodium Salt Bilirubin) OR (delta-Bilirubin) OR (delta Bilirubin) OR (Bilirubin, (15E)-Isomer) OR (Bilirubin, Calcium Salt) OR (Calcium Salt Bilirubin) OR (Salt Bilirubin, Calcium) OR (Calcium Bilirubinate) OR (Bilirubinate, Calcium)
#3	(prognosis [MeSH Terms]) OR (Prognoses) OR (Prognostic Factors) OR (Factor, Prognostic) OR (Factors, Prognostic) OR (Prognostic Factor)
#4	#1 and #2 and #3

### 2.4 Inclusion criteria for study selection

1. Patients diagnosed with HF.
2. Explicit bilirubin level data.
3. The studies must be regarding the discussion on the connection between serum bilirubin level and the prognosis of heart failure.
4. The prognosis was shown in the form of hazard ratios (HR) and 95% confidence intervals (CI) or can be obtained according to demographic data or survival curve.
5. English research papers.

For repetitive documents, we will select documents with larger sample size and more detailed data.

### 2.5 Data collection and analysis

#### 2.5.1 Selection of studies, data extraction and assessment of methodological quality

To ensure the accuracy of the included research, all evaluators will be well trained in accordance with the PRISMA flow chart. Two reviewers will independently screen the title, abstract of the literature to select the eligible studies and eliminate duplicate and unrelated ones. For the studies that are not sure of the certainty, we will further reading the full text to determine whether they will be remained. All the results of selected literatures will undergo cross-check to ensure the accuracy of screening. If there is any inconsistency, the third author will refind the literature and discuss the differences with the 2 reviewers. The data items that will be extracted from the full texts of selected studies include:

1. Titles
2. Author names
3. Year of publication
4. Type of the research
5. Diagnosis of patients
6. The number of patients
7. Follow-up period
8. Prognostic outcome
9. Level of bilirubin
10. Score of NOS

The Newcastle-Ottawa Scale (NOS)^[14]^ will be applied to assess the quality of each selected literature. NOS consists of 3 parameters about the quality evaluation index.^[15]^ The full score of NOS is 9 points, the lowest is 0 points, and scores more than 6 are considered high-quality ones.^[16]^ The evaluation of methodological quality is also completed by 2 independent reviewers, and any disagreements will be solved by an arbiter.

#### 2.5.2 Risk of bias

The 2 authors will use Cochrane Collaboration's tool for assessing risk of bias.^[17]^

#### 2.5.3 Management of missing data

If there is a lack of experimental results, we will try to get in touch with the author of the literature by email to complete the data, if there are unavoidable reasons that lead to failed to fulfill the missing data, we will only process the existing data.

#### 2.5.4 Assessment of heterogeneity

All eligible studies will be tested by Cochran's Q test and I^2^ statistic.^[18]^ Generally, the threshold of obvious heterogeneity will be set as I^2^ > 50%. In general, we choose a fixed-effects model to summarize the data which possess the characteristics of *P* > .05, I^2^ <^ ^50%. For the data with high heterogeneity (*P* < .05, I^2^≥50%), random effects model will be applied.^[19]^

#### 2.5.5 Assessment of publication biases

If more than 10 trials were got, Egger tests and funnel chart will be carried out to check the publication bias.^[20]^ The threshold is set as *P* < .05.

### 2.6 Data synthesis

All statistical tests will be performed using STATA 14.0 software. HR and 95% CI will be calculated for each study to assess the association between bilirubin expression and the prognosis.

For studies where HR and 95% CI are not offered, they will be obtained from the survival curves, and the method adopted from Tierney^[21]^ will be used to calculate HR and 95% CI. The HR and 95% CI of individual study will be finally combined using the STATA software. Pooled HR > 1 indicates poor prognosis for the group of high bilirubin expression, otherwise indicating good prognosis value.

### 2.7 Meta-analysis

The meta-analysis will be implemented by Review Manager (Revman) 5.3.5 software. The software will create forest plots revealing the meta-analysis results which can assess the prognostic utility of bilirubin as an indicator of prognosis in HF. Statistical significance allows us to verify whether the pooled results accept or reject the null hypothesis (in our study, it means if bilirubin shows high/low values, it suggests worse/better survival in HF).

### 2.8 Subgroup and sensitivity analysis

If necessary, we will analyze gender, age, brain natriuretic peptide/N terminal-pro brain natriuretic peptide level, follow-up time to evaluate the influence on prognostic indicators.

Sensitivity check will be conducted by removing one study to evaluate the change on the outcomes and to ensure the sensitivity analysis.

### 2.9 Assessment of the quality of evidence

The Grading of Recommendations Assessment Development and Evaluation (GRADE) Working Group will be assessed to evaluate each major outcome.

### 2.10 Patient and public participation

All the processes of the study do not involve patients and the public.

### 2.11 Ethics and communication

The moral recognition of the study is not necessary, because that the results are based on the published documents.

## 3 Discussion

Although there has been great progress in the treatment of heart failure, there are not many studies on the factors affecting the prognosis of patients with heart failure. There are some literatures that do explore the effect of bilirubin on all-cause mortality in patients with heart failure, but there is currently no meta-analysis on this aspect. Therefore, we want to collect relevant literature through a systematic review to comprehensively analyze the effect of bilirubin on the prognosis of patients with heart failure, so as to obtain a more authoritative research result and provide a reliable research foundation, for clinical doctors to pay more attention to the prognosis of bilirubin in patients with heart failure.

## Author contributions

**Conceptualization:** Yuanhui Hu.

**Data curation:** Huan Wang, Qiulei Jia.

**Methodology:** Huan Wang, Qiulei Jia, Jingjing Shi.

**Supervision:** Yuanhui Hu.

**Writing – original draft:** Huan Wang.

**Writing – review & editing:** Jingjing Shi.
